# Gestational Hypertension and Organophosphorus Pesticide Exposure: A Cross-Sectional Study

**DOI:** 10.1155/2015/280891

**Published:** 2015-08-03

**Authors:** Caterina Ledda, Maria Fiore, Lory Santarelli, Massimo Bracci, Giuseppe Mascali, Maria Grazia D'Agati, Alfredo Busà, Margherita Ferrante, Venerando Rapisarda

**Affiliations:** ^1^Hygiene and Public Health, Department of G.F. Ingrassia, University of Catania, Via Santa Sofia 87, 95123 Catania, Italy; ^2^Occupational Medicine, Department of Clinical and Molecular Science, Polytechnic University of Marche, Via Tronto 10/A, 60100 Ancona, Italy; ^3^Division of Clinical Pathology, Hospital of Taormina, ASP Messina, C/da Sirina, 98039 Taormina, Italy; ^4^Division of Cardiology, Hospital of Lentini, ASP Siracusa, C/da Colle Roggio, 96016 Lentini, Italy; ^5^Occupational Medicine, “Policlinico-Vittorio Emanuele” University Hospital, University of Catania, Via Santa Sofia 78, 95123 Catania, Italy

## Abstract

Hypertension is the most common medical problem encountered during pregnancy, complicating 2-3% of pregnancies. High blood pressure (BP) with diastolic BP ≥ 90 mm Hg and/or systolic BP ≥ 140 mm Hg arising after week 22 of pregnancy and resolving after delivery is defined as gestational hypertension (GHY). The aim of this cross-sectional study was to investigate whether occupational and/or environmental exposure to organophosphorus (OP) pesticide affects GHY. Women at approximately 22 weeks of gestation were recruited. OP pesticide exposure in the first trimester of pregnancy was classified into four categories: no exposure, indirect exposure, domestic exposure, and occupational exposure. Application of the exclusion criteria left 2203 participants (mean age 30.4 ± 11.6 years). Data analysis showed that in women with indirect OP pesticide exposure the incidence of GHY was slightly higher than that in the world population, whereas domestic exposure involved a 7% increase and occupational exposure a 12% increase. Analysis of the pesticides used by participants highlighted a possible role for malathion and diazinon (adjusted OR 1.09 and 1.14, resp.). Further investigation of exposed workers and the general population is clearly warranted given the broad diffusion of OP pesticides and their possible public health impact, maybe by including a wider range of health outcomes.

## 1. Introduction

Hypertension is the most common medical problem encountered during pregnancy, complicating 2-3% of pregnancies [1, 2]. Hypertensive disorders during pregnancy are classified by the Working Group on High Blood Pressure in Pregnancy into four categories: chronic hypertension; preeclampsia-eclampsia; preeclampsia superimposed on chronic hypertension; and gestational hypertension (GHY) [1, 2]. High blood pressure (BP) with diastolic BP ≥ 90 mm Hg and/or systolic BP ≥ 140 mm Hg arising after week 20 of pregnancy and resolving after delivery is defined as GHY [1, 2].

Risk factors include first pregnancy, obesity, high maternal age, preexisting diabetes, renal disease, hypertension, and chronic autoimmune disease [3–7].

Maternal exposure to chemicals has seldom been investigated in relation to hypertensive disorders during pregnancy. Some studies have suggested that lead, cadmium and other metals or elements [8–10], organic solvents [11], air pollution [12–15], and pesticides [16–19] may increase the risk of hypertensive disorders. The effects of organophosphorus (OP) pesticides on hypertension have been investigated in vivo, in the general population and in workers [20–27], but not during pregnancy.

The aim of this cross-sectional study was to investigate whether occupational and environmental exposure to OP pesticides affects GHY.

## 2. Materials and Methods

### 2.1. Subject and Pesticide Exposure

Women at approximately 22 weeks of gestation were recruited by general practitioners or occupational physicians from 2007 to 2013. The study took place in Sicily. Individuals referred by the occupational physicians were recruited to the study by posted study information or direct physician contact. No restriction was placed on the number of participants.

The study protocol was approved by the Catania University Hospital Ethical Committee, and informed consent forms were signed by all participants prior to enrolment.

The enrolment procedure involved a detailed medical history; a structured questionnaire investigating environmental and occupational risk was administered by trained interviewers to gather accurate data on demographics, health habits, and pesticide and other chemical exposures. In order to make a correct classification, specific questions were formulated regarding exclusive OP pesticides. Exposure in the first trimester of pregnancy was classified into four categories: no exposure (women reporting no exposure); indirect exposure (from planting, pruning, weeding, picking, or harvesting); domestic exposure (from pesticide use in the garden or in the house); occupational exposure (from work with pesticides).

The body mass index (BMI) was calculated based on prepregnancy weight referred by each subject.

For BP measurements, using a validated 907 automated digital oscillometric sphygmomanometer (Omron Healthcare Europe B.V., Hoofddorp, Netherlands), participants sat on chairs with a back support. After 20 min acclimatization (room temperature 23.0 ± 2.0°C) a medical doctor placed a cuff around the left upper arm, which rested at the level of the heart, with the stethoscope over the brachial artery pulsation. The mean value of three readings taken over a 20 m interval was recorded.

Participants were considered to have GHY if they had systolic BP ≥ 140 mm Hg and/or diastolic BP ≥ 90 mmHg.

Women with hypertension before pregnancy, anaemia, toxaemia of pregnancy, kidney or heart disease, diabetes, urinary tract infection, metabolic disorders, antiphospholipid antibody syndrome, multiple pregnancies, a BMI < 19 or > 35 kg/m^2^, and those reporting complications during earlier pregnancies and/or deliveries were excluded.

### 2.2. Statistical Analysis

Data were analyzed using SPSS 20.0 software for Windows (SPSS Inc., Chicago, IL, USA). Mean standard deviation (SD) and percentages were used to evaluate descriptive statistics.

Odds ratios (ORs) and 95% confidence intervals (95% CI) for the occurrence of GHY as a function of OP exposure were estimated under conditional logistic regression model. Data were adjusted for maternal age, smoking, alcohol, and BMI. A value of *p* < 0.05 was considered significant.

## 3. Results and Discussion

Of the 2788 pregnant women referred to the study from 2007 to 2013, 157 refused to participate; 107 reported simultaneous exposure to several classes or other types of pesticide; and 321 met one or more other exclusion criteria, leaving 2203 participants whose mean age was 30.4 ± 11.6 years. BP of overall women average was 123 ± 14.1 mm Hg and 82 ± 11.8 mm Hg, for systolic and diastolic, respectively. The only OP used by our population observed are chlorpyrifos, diazinon, malathion, and parathion.

Their exposure data are reported in Table 1.

Hypertension in pregnancy is very common. It complicates almost 10% of all pregnancies and contributes much to maternal and perinatal morbidity and mortality [2]; our data analysis showed that domestic exposure involved a 7% increase and occupational exposure a 12% increase. Analysis of the OP pesticides used by participants highlighted a possible role for malathion and diazinon for GHY (Table 2).

Neither malathion nor diazinon appear to have been investigated in recent studies of the general population or of exposed workers. Earlier studies showed that cardiovascular signs due to acute oral exposure to high diazinon doses include tachycardia [28, 29], hypertension [30, 31], and bradycardia [29, 31].

As regards diazinon, its toxicity is chiefly due to central and peripheral nervous system acetylcholinesterase (AChE) inhibition. AChE is responsible for terminating the action of the neurotransmitter acetylcholine (ACh) at pre- and postsynaptic nerve endings and neuromuscular junctions by hydrolyzing it. AChE inhibition by diazinon is exerted via formation of a stable phosphorylated complex that is incapable of removing ACh. The resulting ACh accumulation at these sites involves constant or anyway excessive cholinergic fibre stimulation at the level of postganglionic parasympathetic nerve endings, neuromuscular junctions, skeletal muscle, as well as CNS cells, giving rise to hyperpolarization and receptor desensitization. Such effects are exerted on end organs innervated by fibres in the postganglionic parasympathetic nerves (e.g., heart, blood vessels, and secretory glands) and induce muscarinic effects that manifest as “miosis, excessive gland secretion (salivation, lacrimation, rhinitis), nausea, urinary incontinence, vomiting, abdominal pain, diarrhea, bronchoconstriction or bronchospasm, increased bronchosecretion, vasodilation, bradycardia, and hypotension” [32], whereas ACh build-up at the level of skeletal muscle junctions and sympathetic preganglionic nerve endings gives rise to nicotinic effects that manifest as “muscle fasciculation, weakness, mydriasis, tachycardia, and hypertension” [32].

Cardiovascular effects were noted in nearly all reported cases of malathion poisoning [33–36]. Admission signs and symptoms generally included bradycardia and low BP, as in vagal stimulation. Several patients also had atrioventricular conduction disturbances within a few days of pesticide ingestion [33, 34]. The doses ingested in these cases ranged from 214 to 2,117 mg/kg. In contrast, Choi et al. [37] reported abnormal emergency room electrocardiogram and chest X-rays in a woman who ingested approximately 1.071 mg/kg. Tachycardia may be the result of cholinergic stimulation of parasympathetic and sympathetic autonomic ganglia. Administration of gavage doses of 390 mg kg/day (unspecified purity) for 1-2 weeks caused focal haemorrhage in the heart of rats [38]. A single gavage dose of 1.950 mg/kg (95% pure), the only malathion dose tested, caused congestion and haemorrhage in the heart of male Wistar rats 2 days after dosing; females were not tested [39].

Based on our findings and the literature data correct exposure information is clearly required for OP pesticide use by pregnant women, whatever the exposure level. In fact two studies of other classes of pesticides confirm that the agent is passed from mother to fetus [17, 40]. In addition pesticides are known to play a role in inflammation processes [41, 42].

## 4. Conclusions

This study suggests that the OP pesticides malathion and diazinon may be associated with GHY, although the literature reports limited evidence for this notion. Further investigation of exposed workers and the general population is clearly warranted given the broad diffusion of OP pesticides and their possible public health impact, maybe by including a wider range of health outcomes. The level of exposure should also be investigated in greater detail via biological indicators of damage and inflammation processes.

## Figures and Tables

**Table 1:** Characteristics of participants.

	No exposure	Indirect exposure	Domestic exposure	Occupational exposure	Total
Subject *n* (%)	**582** **(26% of total)**	**534** **(24% of total)**	**613** **(28% of total)**	**474** **(22% of total)**	**2203**
Age					
18–22 y	98 (17%)	94 (18%)	78 (13%)	69 (15%)	339 (15%)
23–27 y	127 (22%)	102 (19%)	114 (18%)	63 (13%)	406 (18%)
28–32 y	136 (23%)	115 (21%)	145 (24%)	103 (22%)	499 (23%)
33–37 y	120 (21%)	121 (23%)	153 (25%)	128 (27%)	522 (24%)
38–42 y	101 (17%)	102 (19%)	123 (20%)	111 (23%)	437 (20%)
Education					
Secondary	196 (34%)	124 (23%)	135 (22%)	273 (58%)	728 (33%)
High school diploma	286 (49%)	286 (54%)	236 (39%)	147 (31%)	955 (43%)
Degree	100 (17%)	124 (23%)	242 (39%)	54 (11%)	520 (24%)
Body mass index					
<24 kg/m^2^	185 (32%)	167 (31%)	126 (20%)	102 (22%)	580 (26%)
25–30 kg/m^2^	265 (45%)	243 (46%)	274 (45%)	275 (58%)	1057 (48%)
>30 kg/m^2^	132 (23%)	124 (23%)	213 (35%)	97 (20%)	566 (26%)
Smoking habits					
No	247 (42%)	223 (42%)	278 (45%)	264 (56%)	1012 (46%)
Until she knew of her pregnancy	272 (47%)	264 (49%)	276 (45%)	136 (29%)	948 (43%)
Yes, during pregnancy	63 (11%)	47 (9%)	59 (10%)	74 (15%)	243 (11%)
Alcohol drinking habits					
No	241 (41%)	238 (45%)	247 (40%)	172 (36%)	898 (41%)
Until she knew of her pregnancy	243 (42%)	214 (40%)	269 (44%)	216 (46%)	942 (43%)
Yes, during the pregnancy	98 (17%)	82 (15%)	97 (16%)	86 (18%)	363 (16%)
Gestational hypertension	26 (4%)	33 (6%)	41 (7%)	55 (12%)	155 (7%)

**Table 2:** Association between use of organophosphorus pesticides and gestational hypertension.

	Gestational hypertension			
	*n*	% exp	aOR	95% CI
No exposure	26	—	1.00	
Chlorpyrifos	25	20	1.03	0.86–1.08
Diazinon	30	23	1.09^*∗*^	1.03–1.16
Malathion	48	37	1.14^*∗*^	1.08–1.19
Parathion	26	20	1.02	0.78–1.19

% exp = proportion of participants with gestational hypertension actually exposed to the agent.

aOR = effect estimates adjusted for age, smoking and alcohol drinking habits, and BMI.

^*∗*^ *p* value < 0.05.
